# Surgery

**Published:** 1861-07

**Authors:** John H. Packard

**Affiliations:** Philadelphia


OR, BI-MONTHLY NOVELTIES IN MEDICAL, PATHOLOGICAL, SURGICAL, OBSTETRICAL, PHYSIOLOGICAL, AND CHEMICAL SCIENCE.

### SURGERY.

BY JOHN H. PACKARD, M.D., OF PHILADELPHIA.

I.—*Statistics of Amputation at St. George's Hospital during Seven Years.* By G. F. COOPER and T. HOLMES, formerly Surgical Registrars to the Hospital.

THESE statistics comprise 149 amputations: 76 of the thigh, 40 of the leg, 22 of the arm, and 11 of the forearm. Death ensued in 41, or 27·6 per cent. of the whole number.

Of the 76 thigh cases, 25, or 32·9 per cent., terminated unfavorably; of the 40 leg cases, 13, or 32·5 per cent.; of the 22 arm cases, 3, or 13·7 per cent.; of the 11 forearm cases, all did well.

*Amputations of the Thigh.*—Of these, 13 were for injury, and the remaining 63 for various other causes. Of the former, 9 were primary, with 5 deaths,—2 from pyæmia, 2 from shock, and 1 from other injury. Two of the 4 secondary operations were unsuccessful, the patients dying of pyæmia. Hence 7, or 53 per cent., of the 13 cases ended fatally.

Fifty-one of the remaining 63 cases are classified as "pathological," with 13 deaths (25·6 per cent.;) 12 as "of expediency," with 5 deaths (41·6 per cent.;) 18, or 28·5 per cent., therefore, in the whole 63. Of the 51, 8 amputations were performed for abscess in the knee-joint; 5 of the patients died,—1 of phthisis and sloughing of the stump, and 4 of pyæmia. Thirty-five were for chronic affections of the knee; of these, 5 died,—2 of pyæmia, as proved by post-mortem examinations; in 2 others pyæmia was indicated by the symptoms during life; 1 from phthisis and sloughing of the stump. Of the remaining 8 cases, 2 were for malignant disease, 2 for gangrene after ligature of the femoral artery, 2 for necrosis, 1 for arterial hæmorrhage in phagedæna, and 1 for necrosis and abscess after excision of the knee; 3 died,—1 of exhaustion, 1 of secondary hæmorrhage, and 1 of pyæmia.

Of the 12 "amputations of expediency," 9 were for tumors, 2 for loss of skin after diffuse cellular inflammation, and 1 for old contraction of the knee with atrophy of the leg; of these, 5 died,—3 of pyæmia, 1 of exhaustion, and 1 of secondary hæmorrhage.

*Amputations of the Leg.*—Of these 9 were traumatic, 6 being primary and 3 secondary; 4 of the 9 died,—2 from pyæmia, 1 from secondary hæmorrhage, and 1 from exhaustion.

Twenty-seven of the remaining 31 were "pathological," 3 for acute disease of the ankle-joint, with 1 death; 24 for chronic disease of the bones of the foot and leg, with 8 deaths,—4 from pyæmia, 3 from strumous disease of internal organs, and 1 from exhaustion. Of the 4 "amputations of expediency," which all did well, 2 were for painful stump, and 2 for tumors.

*Amputations of the Arm.*—Of the 22, 14 were for disease, with 2 deaths; 8 for accident, 6 primary, with 1 death, and 2 secondary. Ten of the 14 were for disease of the bones or of the elbow-joint; 3 of the remaining 4 were on account of the effects of burn, and 1 (at the shoulder) for malignant tumor of the humerus. All the deaths were from pyæmia.

*Amputations of the Forearm.*—These were 11 in number, 4 primary, (probably for injury,) 6 for disease of the hand, and 1 for a tumor. They all ended favorably.

REMARKS.—The reporters adopt, as has been seen, the classification employed by Mr. Bryant in a paper in vol. xlii of the Medico-Chirurgical Transactions; they, however, object to it as somewhat arbitrary. The "pathological" amputations amount to 94, of which 24, or 25·5 per cent., died; amputations "of expediency" to 21, of which 5 died, or 23·8 per cent.; primary amputations to 25, of which 9, or 36 per cent., died; and secondary to 9, of which 3, or 33·3 per cent., died. These figures, so far as they go, do not bear out Mr. Bryant's statement, that amputations "of expediency" are much more fatal than "pathological," and secondary than primary. But the list is not long enough to justify any very positive deductions.

*Comparative Mortality of Circular and Flap Operations.*—Of the 149 operations included in the table, 46 were by the flap method, and 97 by the circular; 3 were not noted as to this particular, and 3 by Syme's method are not counted. Of 74 amputations of the thigh, 52 were circular, 15 of which died, showing a mortality of 28·8 per cent.; 22 were flap, and 10 died, showing a mortality of 45·4 per cent.

The shortest period between the operation and the patient's discharge from the hospital was, in the circular, 23 days, and in the flap 24; the longest, in the circular 126, and the flap 105 days; the average period of cure in the circular 60 days, and in the flap 42.

Of 37 amputations of the leg, 21 were circular, of which 3 died,—a mortality of 14·2 per cent.; 16 were flap, and 8 died,—a mortality of 50 per cent. With respect to the period of their stay in the hospital, the shortest period was in the circular operations 19, and in the flap 20 days; the longest, in the circular 120 days, and in the flap 94; the average, in the former 49, and the latter 49 days.

Of 22 amputations of the arm, 17 were circular, of which 2 died,—a mortality of 11·8 per cent.; 5 were flap, of which 1 died,—a mortality of 20 per cent. The shortest period in hospital was, in the former 20, in the latter 26 days; the longest, in the former 96, in the latter 70 days; the average, in the former 41 days, in the latter 51.

Of 10 amputations of the forearm, 7 were circular and 3 flap; none died. The shortest time in hospital was, in the former class 20, in the latter 17 days; the longest, in the former 142, in the latter 50; the average of the former 44, of the latter 29 days.

Hence it appears that out of 97 circular amputations of all kinds, 20 died,—a mortality of 20·6 per cent.; while out of 41 flap 19 died,—a mortality of 41·3 per cent., or just double that of the circular; and this excess of mortality of the flap over the circular prevailed in all classes of amputations. More extensive data would, however, be required to warrant any conclusion in regard to this.

*Influence of Sex.*—Of the 149 patients, 118 were males, and 31 females. The mortality was 34, or 28·8 per cent., of the former, and 7, or 21·9 per cent., of the latter. In view of the facts that males are far more frequently subjected to traumatic amputations than females, (32 to 2 in the foregoing list,) and that these are more dangerous than those for disease, we may perhaps infer that females bear the operation worse than males.

*Influence of Age.*—It would seem that the greatest success attends amputation in those below 15 years of age, the least in those between 25 and 60; above this latter age we have not sufficient data.

*Causes of Death.*—The total number of deaths was 41; the assigned causes as follows: Pyæmia in 24 cases, or 58·5 per cent.; exhaustion (without hæmorrhage) in 7, or 17 per cent.; exhaustion, with secondary hæmorrhage, 4, or 9·7 per cent.; visceral disease, in 4, or 9·7 per cent.; diffuse inflammation and gangrene, in 1, or 2·4 per cent.; other injuries, in 1, or 2·4 per cent.; in 9 cases no post-mortem examinations were made.—(*Medical Times and Gazette*, April 6, 1861.)

II.—*On Cases of Recovery from Traumatic Tetanus.* By MR. JONATHAN HUTCHINSON.

Mr. Hutchinson has collected in a tabular form the London hospital reports of cases of this kind, in order to facilitate their comparison. He refers to a previous series, having special reference to the prognosis and treatment of the disease, upon which some general remarks may be found in the *Medical Times and Gazette* for June 17, 1854. We must be content here with presenting Mr. Hutchinson's comments merely.

In his table, most of the cases show an unusually long interval between the receipt of the injury and the invasion of tetanic symptoms; seldom less than ten days, and generally much over that time. "Those in which it was shorter are, for the most part, precisely those in which there is reason to doubt whether the disease was really excited by the asserted injury or not. As it regards cases in which tetanic symptoms supervene in a well-marked form within ten days of a *bonâ fide* injury, there is as yet but little evidence of a hopeful nature on record. The longer the interval (or period of incubation) the better the prognosis. Among other circumstances and symptoms of good omen, we may briefly mention the following, which we place in the order of their estimated value: 1. If the symptoms develop themselves slowly. 2. If there is little or no tendency to pharyngeal spasm. 3. If the pulse does not become greatly quickened. 4. If the skin remains cool and the bowels respond to mild purgatives."

The table shows success with very various plans of treatment. Mr. Hutchinson sums up his conclusions from these and other cases as follows:—

"1. The chief objects in view are three: to mitigate the force of the local irritation to which the disease is due; to sustain the patient's strength by food; and lastly, by procuring sleep, to allow the nervous system an opportunity of regaining its wasted powers.

"2. If the case be seen in the very onset, and if the injured part be a finger or toe, it is desirable at once to amputate, whatever may be the local condition. If the injury has been severe, and the part has passed into a state in which, whether from sloughing or otherwise, its recovery is doubtful, it is desirable to amputate at any stage of the disease, or even if one of the extremities be the part involved.

"3. The injured part should be poulticed, and the limb above it wrapped in lint soaked in laudanum or chloroform.

"4. The patient should be put in a room with but one attendant, and the strictest quietude should be insisted on.

"5. If the patient has been accustomed to it, he should be allowed to smoke.

"6. The bowels should be well cleared out by croton oil or other efficient purgative.

"7. If the skin be very hot, the pulse jerking, and the tongue red and dryish, the surgeon may be justified in combining small doses of calomel with the narcotic he may have selected for employment.

"8. A free allowance of beef-tea, milk, eggs, and similar articles of concentrated fluid nutriment should be given, more especially in the later stages of the complaint.

"9. As long as the patient is able to take food and to obtain periods of comparative quiet and freedom from pain, the use of anæsthetic inhalations is not desirable. Great advantages may, however, be obtained from them if he be unable to open the jaw sufficiently to admit of taking food, or if the tetanic spasms are without remission. Ether appears to have stronger facts in its recommendation than chloroform.

"10. One or other form of narcotic—opium, Indian hemp, belladonna, or woorara—should be freely used. There is no very decisive evidence as to the advantage of any one of these over the others. Respecting the Indian hemp and the woorara, the difficulty often encountered in obtaining them in a state of reliable activity will often be an obstacle to their employment.

"11. Excepting possibly in the per-acute cases, the free use of quinine appears to be desirable. If given in large doses it generally reduces the frequency of the pulse, and in some cases a mitigation of the tendency to spasm has attended its influence. The rapid induction of cinchonism is a measure well worthy of a fair trial."—(*Medical Times and Gazette*, April 6, 1861.)

### III.—*On Pyæmia.* By PROF. ROSER, of Marburg.

The following observations form a brief abstract of some papers on this subject, by the above-named author.

*The Specific Nature of the Disease.*—We are indebted to the obstetrician rather than to the surgeon for any progress which has been made in our knowledge of pyæmia, he having established the miasmatic character of the pyæmia of puerperal women, and its identity with the pyæmia of the wounded. The doctrine of "Surgical Fever," so ably expounded by Simpson, has, however, made but little way in Germany, Virchow's views on thrombosis there predominating, as the doctrine of phlebitis formerly did in France. Professor Roser shows that neither Hunter's theory of phlebitis, Rokitansky's disease of the blood-vessels, nor Virchow's thrombosis afford any sufficient explanation of pyæmia, conditions being assigned as causes, which are mere concomitants or effects. The attempt to explain its occurrence by the fact of the absorption of ill-conditioned pus also fails; for, although various analogous circumstances are producible by such absorption, these differ much from those of pyæmia, and may be exposed by the word septæmia. The two conditions may, indeed, be combined, and we may have a septic pyæmia, just as we may have a septic variola or scarlatina. If pyæmia be followed out through its various modes of manifestation, it will be found to exhibit a marked similarity to typhus and other zymoses; and just as in the case of these zymoses, while sometimes it appears epidemically, and as the result of contagion, at others it arises spontaneously, without the prior presence of pus. This fact has long been known in lying-in hospitals, and careful observation will easily detect similar cases in surgical wards. Stromeyer has observed a whole series of such cases, and similar ones have come under the author's notice. The explanation of the occurrence of sporadic cases of pyæmia may be difficult, but it is no more so than is the explanation of sporadic typhus or cholera, or other zymoses.

*Forms of Pyœmia.*—Professor Roser confines his attention to some of these which have attracted but little notice. 1. *Pyœmic Febricula:* Just as during the prevalence of typhus, we find patients here and there exhibiting but slight symptoms, so in hospitals infected with pyæmia, a mild form of the disease is observable which may be termed febricula. It has been but little noticed, as was indeed to be expected, by those who were on the look-out for phlebitis, sepsis, or thrombosis, as the initial phenomenon of pyæmia; but the author instances cases in his own and in Stromeyer's practice. In lying-in hospitals the febricula is termed "milk fever"—a term of doubtful propriety, seeing that the affection is observed sometimes in almost all the inmates, and at others in none of them. 2. *Pyœmic Erysipelas:* When, in a patient suffering from pyæmia, erysipelas appears, the natural conclusion is that the pyæmic blood-disease has localized itself in the skin, just as it might have done in the pleura or in a joint. When, however, in a subject of erysipelas symptoms of pyæmia appear, the question may arise, whether during the erysipelas the blood-poison has been developed, whether the pyæmia has become added as a second special process of disease, or whether the erysipelas itself was only the first manifestation of the pyæmic condition. Lastly, erysipelas and pyæmia may coexist, and although they may not often be met with in the same patient, they are frequently found prevailing among different patients in the same ward. The author's conclusion is, that hospital erysipelas is a consequence of pyæmic infection, and its localization in the skin, although he admits that it is doubtful whether another variety of blood-poison may not also give rise to it. 3. *Pyœmic Diarrhœa:* This affection, well known to clinical observers, has obtained but little notice in hand-books. There are cases in which no other symptom except the diarrhœa is present, but the author still regards these as pyæmic, occurring as they do simultaneously with other cases in which the diarrhœa has only been the first of the whole series of symptoms. When this diarrhœa is combined with the pyæmic erysipelas, the disease exhibits striking and rapid contagious properties; and in hospitals in which precautions against contagion are not taken, this hospital epidemic diarrhœa, though little spoken of, is of frequent occurrence.

*Therapeutics.*—Although the incurability of pyæmia is no longer believed in, practitioners in general are not aware of the number of recoveries which really do take place; and that even when excluding the slighter cases above alluded to, and admitting only examples of well-marked pyæmia. The number of recoveries has increased in proportion as the essential conditions of fresh air and a good diet have been appreciated. Supporting the patient's strength by means of wine, has replaced the former mischievous antiphlogistic treatment; and, indeed, judging from the use made of alcohol in England, there is danger of the opposite extreme being fallen into. Quinine, though usually of no great utility, may in some cases be a valuable adjuvant; and morphia is an invaluable remedy, serving not only to check diarrhœa, abate pain, and diminish danger of peritonitis, etc., but also to tranquilize the excited and delirious patient. The most important agent in the treatment, however, is a frequent renewal of fresh air, and the removal of all objects likely to pollute it.

*Prophylactics.*—Under this head the author lays great stress upon simplicity in dressing wounds, observation of the strictest cleanliness, and checking the decomposition of pus by cold and chlorine applications. When the spread of pyæmia is to be guarded against, instruments and nursing appliances should not be used in common, no autopsy should be performed by those attending on the sick, and the surgeon, visiting his cases of pyæmia last, should change and ventilate his clothes before seeing other patients. In all cases of ill-conditioned suppuration at Marburg, weak chlorinated water is employed, and after waiting on such patients, the nurses carefully wash their hands in the same fluid. Finally, Dr. Roser protests against the erecting of hospitals with large surgical wards, unaccompanied by means for isolating the subjects of pyæmia. Small hospitals, of even a very faulty construction, give rise sometimes to a less mortality than some magnificent structures in which the patients are assembled together in large numbers.

*Juridical Relations.*—Under this head the author discusses the question which may come before the surgeon in a court of law, viz.: whether a fatal pyæmia following an injury not in itself necessarily fatal, should be regarded as an essential condition of such injury, or as an accidental and superadded circumstance; the exact determination of this point modifying in the German courts the amount of punishment to be awarded. He cites cases in which the pyæmic complication has been altogether overlooked, or has been wrongly interpreted, to the detriment of the accused. He refers also to two other conditions to which the same considerations apply, viz.: diffuse inflammation and tetanus. Diffused inflammation, after wounds of tendons, etc., so often observed in hospital, and so seldom in private practice, should not be attributed to the nature of the local injury, but to the presence of miasmata. In fact, it is of a pyæmic nature. With respect to tetanus, we know nothing concerning its causal connection with wounds, and when questioned juridically, we should avow such ignorance. The author himself is disposed to regard it as a zymotic affection, not only because no causal connection with the wound can be made out, but from its analogy to hydrophobia, which is a zymotic affection, and from its disposition to prevail as an epidemic or endemic.—(*Brit. and For. Med.-Chir. Review*, April, 1861; from the *Archiv für Heilkunde*, Jahrg. i.)

IV.—*On the Relative Safety and Efficiency of Ether and Chloroform as Anæsthetics.* By FREDERICK D. LENTE, M.D., of Cold Spring, N. Y.

Dr. Lente refers to some statistics published a few years ago by Dr. Kidd, of London, from which it might be inferred that ether was quite as dangerous an article as chloroform. This idea, however, is not borne out upon a careful examination of the data; and it will not probably commend itself to any one much in the habit of administering ether. Hospital experience, civil and military, has furnished no recorded instance of death from this anæsthetic; the French authorities seem even to differ as to the results of the observations of the army surgeons in the Crimean war, with regard to the danger from chloroform. Physiological experimenters have noted that animals are much oftener fatally affected by chloroform than by ether. Moreover, when evil effects do manifiest themselves, those from the latter agent are much more manageable than those from the former.

Dr. Lente suggests one important idea, viz.: that the mixture of chloroform with ether only augments the danger, by diminishing the caution of those who administer the anæsthetic, while the greater volatility of the ether causes it to evaporate so fast that the chloroform may be left nearly pure for inhalation.

He advises the giving of ether with *as little admixture of atmospheric air as possible*, in order that it may take effect quickly and without waste; averring that this plan adds nothing to the risk. All draughts of air should, in his opinion, be excluded, as delaying the induction of the desired state, which should be brought about, he thinks, in five or six minutes in adults, and in a much less time in children.—(*American Journal of the Med. Sciences*, April, 1861.)

V.—*Removal of a Ball by means of the Trephine, Twenty-two Months after its Penetration into the Cranium.* By M. JOBERT.

Jules Gustin, aged twenty-one, was admitted into the Hôtel-Dieu, on the 19th of February, 1857. Forming one of a force posted in front of the Malakoff tower, April 8th, 1855, he was struck by a ball in the forehead, the projectile, prior to penetrating the cranium, having come in contact with the external surface of the vizor, leaving a semilunar depression as it passed over the edge of this. The man immediately fell from the parapet into the trench, a depth of seven feet, and remained unconscious in the ambulance for twenty-four hours. At the end of a week he was sent to the military hospital at Constantinople, where he stayed during four months. He then requested permission to return to the Crimea, and did so, in spite of the suppuration, which had never ceased. He fought at Traktir, August 16th, 1855, and returning to Paris, in December, he remained for six months longer in the regiment. He was, however, unfitted for active service, being obliged to pass a considerable time in hospital. The symptoms continued much the same from the first, and consisted in a sensation of heaviness of the head, an uncertainty in the attitudes, and a feeling, when stooping, as if the forehead were separating from the head, suppuration always persisting.

On his admission into the Hôtel-Dieu, a clean, circular aperture, about the size of a franc, was observed in the centre of the forehead. Passing the finger around the circumference of the opening, "osseous granulations, partial ossifications formed by the periosteum," could be felt, and on the introduction of a probe to the bottom, a hard, resisting, metallic body was recognized. After a crucial incision had been made, the aperture caused by the ball being found too small to admit of its extraction, a circle of bone was removed by means of the trephine. The ball was now extracted, and some hard, blackish blood-clots removed. The movements of elevation and depression of the dura mater, isochronous with the pulse, were plainly perceptible. The projectile proved to be a leaden ball weighing 25 grammes (375 grains.) Its surface was smooth and spherical over only a small portion of its extent, the remainder being rough and irregular. The details of the recovery need not be pursued, it sufficing to say that this was complete, the man having been seen in perfect health as late as October, 1860.

M. Jobert calls attention to the fact of the ball having remained for so long a period in contact with the dura mater without inducing any inflammatory action. He also lays stress upon the circumstance of his having, after the completion of the operation, introduced the flaps formed by the crucial incision into the accidental opening. To this he attributes the non-occurrence of necrosis or osseous exfoliation usually observed after trephine operations, when the parts are exposed to the air. Here, such exposure was prevented, immediate union of the raw flaps with the bleeding osseous surface being secured. The isochronous pulsations continued for a time visible, but they became more and more obscure as the tissues covering the aperture augmented in thickness.—(*Brit. and For. Med.-Chir. Review;* from the *Comptes Rendus*, 1861, No. 6.)

VI.—*Exostosis of the Left Transverse Process of the Seventh Cervical Vertebra, surrounded by Blood-vessels and Nerves; Successful Removal.* By Mr. Coote.

This tumor, which occurred in the person of a servant girl, aged twenty-six years, had been growing since her infancy, and was about the size of a large walnut; it pushed the subclavian artery forward, and caused pain in the arm and elbow; pulsation was wanting at the left wrist. The girl was otherwise in good health when she applied for treatment at St. Bartholomew's Hospital.

Upon consultation, the removal of the growth was decided to be advisable; and the girl having been placed under the influence of chloroform, the operation was performed without difficulty, although of course with caution. Mr. Coote found the tumor, which he regards as a development of a cervical rib, to be attached to the first dorsal rib also; and was obliged to take away the mass in separate portions.

The case subsequently did well.—(*Lancet*, April 13, 1861.)

VII.—*A Case of Gunshot Wound, the Ball being found in the Substance of the Wall of the Heart, Eighteen Years afterward.* By G. B. BALCH, M.D.

In June, 1842, a boy named John Kelly received a ball in his right shoulder. This ball had passed through three inch-boards before it struck him; it entered through the border of the trapezius muscle, an inch and a half or two inches from the acromion process, and a surgeon who was called found it lodged behind the inner third of the clavicle, but was unwilling to attempt its extraction. In six weeks the boy was at work again.

Four years afterward he had a dangerous attack of pneumonia, with severe and irregular palpitation of the heart; and from that time symptoms of organic disease of the heart were always present.

Strabismus, and sometimes inflammation of the right eye, existed from the time of the accident to that of his death, which took place in June, 1860, as the immediate result of cold taken in bathing. During his last illness, he had severe cardiac disturbance, dysphonia, and pains in the shoulders and arms. His right arm became purple and cold.

At the autopsy, "the right subclavian artery was filled with ossific matter at the thyroid axis; the other arteries were healthy. The right internal jugular and subclavian veins were enlarged; the right external jugular was closed near its union with the internal. The upper lobe of the right lung was congested. There were no tubercles in the lungs, but there was considerable pleuritic adhesion. The heart was enlarged, and undergoing fatty degeneration. The pericardium was very adherent; so much so that I could not separate it from the heart, without cutting either one or the other. At the lower part of the right ventricle I felt a hard lump. I passed my finger into the right ventricle, and found the lump to be in the wall of the ventricle, near its lower part. When cut with my scalpel from the outside down upon the lump, I found it to be a leaden bullet, slightly flattened."—(*Glasgow Medical Journal*, April, 1861; from the *American Medical Monthly*, September, 1860.)

VIII.—*On Gunshot Wounds produced during the Loading of Artillery.* By DR. CORTESE.

Dr. Cortes having related five cases of gunshot wounds received by artillerymen while loading their guns, and pointed out the peculiar severity of such injuries, thus sums up his subject: 1. No other blow of a projectile imparts so great an amount of commotion to the entire limb. The state of muscular contraction prevailing at the time constitutes a kind of solidarity between the hand, forearm, and arm, which is the chief and necessary cause of this commotion. 2. This circumstance compels the surgeon to direct his attention to the entire limb, whatever amount of lesion may be manifest in the hand. A neglect of this precept may lead to gangrene gaining possession of a large portion of the limb, or to a generalized suppuration, while a diminished power of reaction in the injured parts may give rise to purulent infection, or render amputation useless. 3. In all those cases in which the hand is severely torn, its disarticulation, or even the amputation of the forearm, is insufficient to secure recovery. The surgeon's knife penetrates into infiltrated tissues, more or less destroyed in their intimate structure in consequence of the concussion they have been subjected to. So that independently of the fracture of the bone, and of the possible disjunction of the articulations of the ulna, the success of the reparative process would be very problematical. In such cases the arm should be amputated. 4. The sooner amputation is performed, the greater is the probability of a favorable result. 5. The rapid and very extensive tumefaction of the limb constitutes a sufficiently certain criterion of the severity of the derangements which are propagated along its whole extent. When fractures are not detected in the diaphysis of the bone, some lesion of contiguity or continuity in the ulnar articulation must be suspected. 6. When the lesion does not seem severe enough to call at once for amputation, we must always be prepared for secondary occurrences which will unfit the limb for its functions. (In two of the author's cases paralysis of the limb remained.) Still, conservative treatment should in such cases be attempted.—(*Brit. and For. Med.-Chir. Review*, April, 1861; from *Omodei Annali Universali di Medicina*, vol. clxxiv.)

IX.—*On Resection of the Hip-joint in Cases of Coxalgia and of Gunshot Wounds.* By M. LEFORT. (Paper read at the *Académie de Médecine*, Paris.)

The author sums up his paper as follows:—

In view of all the indications and contra-indications of resection of the hip-joint, we may say that it should not be considered as a mode of treatment in coxalgia; it is an exceptional operation, which may and should be performed in grave cases only, where, whether from the extent of the local lesion, or from the impairment of the general health induced by this, the patient's life is seriously compromised, and ordinary measures afford little or no hope of a cure. It is for the surgeon to decide in each case as to the propriety of the operation; absolute rules cannot be laid down.

Coxalgia, when unaccompanied by abscess and caries, when it preserves the form which may be called fungous arthritis, does not call for resection. Prolonged rest, with appropriate general and local treatment, quite frequently effect a cure. The anchylosis which occurs in such cases does not prevent the limb from being of use in walking, if only care has been taken to keep it in a straight position.

There may be ground for doubt as to the propriety of the operation in cases where there is abscess in the joint. The affection is apt then to be of long continuance; openings occur at different points near the articulation, giving exit to a sero-purulent discharge, which, by its nature and its abundance, exhausts the patient.

Almost always, there is a spontaneous luxation; if a cure is obtained before the bones are seriously affected, it is but tardy, and leaves the limb shrunken, deformed, often flexed upon the pelvis, and too short to be useful in walking, so that crutches are necessary. But although in this form of disease the erosion and destruction of the cartilages have almost always determined alterations in the bone, at least in the rim of the cotyloid cavity, resection is not indicated unless the patient's life is seriously threatened.

But if abundant suppuration, and a prolonged confinement in bed have so reduced the strength that the system seems unequal to the task of expelling and making up for the carious or necrosed portions of bone; if the patient's life is in danger; if the bones are affected to a sufficiently great extent; if, above all, spontaneous luxation has taken place, resection is fairly indicated. The enlargement of the cotyloid cavity, so far from being a contra-indication, makes the operation necessary, for by permitting the removal of the carious portions of the ilium, it enables us to check the progress of the malady, and to prevent the formation of abscesses around the pelvis, which are so apt to destroy life.

But doubt often exists as to the extent of the deep-seated disease; the irregularity of the fistulous passages prevents the probe from reaching the bone so as to detect the caries with certainty. It is then that a sort of exploratory operation, often practiced in cases for resection, may be usefully resorted to. The abscesses are freely opened; contiguous or even distant sinuses are laid into one, so that the affected parts may be rendered more easily accessible. If caries does not exist, this operation makes matters no worse; if it does, resections may be intelligently practiced, without fear of subjecting the patient to unnecessary dangers by a rash operation. Resection may be performed even for a necrosis of small extent, and although a spontaneous cure is possible, if the limb has been kept in such a position that its deformity would render walking, even with crutches, a matter of difficulty.—(*Gazette Médicale de Paris*, December 8, 1860.)

X.—*Case of Popliteal Aneurism cured by Forcible Flexion.* By AUGUSTIN PRICHARD, ESQ., Surgeon, Bristol.

John Gough, aged thirty-four, came under Mr. Prichard's care at the Bristol Royal Infirmary, for a pulsating tumor of the left ham, which had existed rather more than two months. The swelling was large enough to cause bulging of the muscles at the inner side of the knee, its transverse diameter being greater than its vertical; its pulsations were very marked, and the leg œdematous, weak, and painful.

It was easy, by means of a leather anklet, with a band attached, to flex the leg strongly upon the thigh, and thus to check the current of blood through the aneurism; but the swelling was so great that this position soon became irksome, and therefore a tourniquet was applied, to be tightened whenever the patient found himself obliged to straighten the knee. By this means an absolute cure was effected in twenty-five days, and although there was some little difficulty at first in overcoming the contraction of the soft parts around the joint, it was soon done away with, and the limb became perfectly sound and serviceable.—(*British Medical Journal*, March 30, 1861.)

XI.—*Fracture of Patella treated successfully by means of Malgaigne's Hooks.* By DR. PACKARD, of Philadelphia.

This case was one of simple transverse fracture, the lower fragment being very small; the patient could not give an exact account of the injury, but she missed a step in going down stairs, and did not know of her having sustained any blow on the part. The hooks were inserted on the eighth day of the treatment, and retained in place for thirty-one days; they excited no inflammation, and caused no pain whatever after the first few hours. A perfect cure was obtained, so that the patient was able to walk in the street in nine weeks.

This apparently formidable, but really innocent and useful instrument, is made and employed as follows: It is composed of two plates of steel, three-quarters of an inch in width and not quite one-eighth of an inch in thickness. Each of these plates is at one extremity recurved downward into two very sharp hooks an inch and a half long, the points of which are parallel with the plates themselves. The upper plate is two inches and a half in length before the curve of the hooks becomes decided; the lower one is shorter than this by an inch. Projecting up from the lower plate, about three-eighths of an inch from the notch between the hooks, is an upright, which runs in a slit rather more than an inch and a half long in the upper plate; at the extremity of this slit is another upright, fixed in the upper plate, and these two uprights are pierced by a screw which runs parallel to the plates, forcing them, and therefore the points of the hooks, together. The screw is squared at one end, so as to be turned by means of a key.

The lower pair of hooks are to be inserted first, the skin being drawn slightly downward; they should catch the edge of the tip of the lower fragment on either side of the ligamentum patellæ. Next the upper plate is slipped on to the lower, (which is turned up for the purpose,) and its hooks forced in so as to pierce the skin at right angles, and engage the points in the upper edge of the upper fragment of the bone. The screw is now passed through the uprights, and turned until the fragments are brought as firmly together as may seem safe. When screwed home, the opposing points are rather less than an inch and a half apart.—(*American Journal of the Medical Sciences*, April, 1861.)

### XII.—*On the Reproduction of Necrosed Bones after Resection.* By M. MAISONNEUVE.

M. Maisonneuve is said to have laid before the Academy of Sciences, at its last meeting, the notes of an interesting case of conservative surgery. In the month of August, 1855, a young man consulted this practitioner in regard to an extensive necrosis of the tibia of the right leg. The limb presented a deplorable aspect, being triple its natural size, and furrowed by deep ulcers, through which the bone could be perceived to be necrosed during its entire length. The disease had commenced after a fall, which occurred two years previously, and the excessive suppuration had occasioned such constitutional disturbance as to threaten life. The most eminent surgical authorities of Paris had declared that only amputation of the thigh could save life, and that the limb itself was beyond hope. M. Maisonneuve determined, however, to test the powers of the periosteum for repair, by performing a sub-periosteal resection of the entire tibia; and the patient readily consenting, this course was at once adopted. Chloroform having been given, an incision fourteen inches in length was made in the periosteum, and the necrosed mass, which did not involve the articular faces of the bone, was removed entire. The wound healed kindly, and by the fortieth day the patient was able to walk, with the aid of crutches. Eventually, the whole bone was reproduced, so that the leg differs from its fellow in no respect except in the cicatrix along its anterior surface.—(*Paris Correspondence of the London Lancet,* April 6, 1861.)

[We have quoted this case because so far as we know it is the first, and it certainly is the most successful, case reported of the application on a large scale of our knowledge of the regenerative powers of the periosteum. If the account be not too highly colored, the result was favorable in a most extraordinary degree.—P.]

XIII.—*On the Means for Preventing Caries of the Petrous Bone and the Formation of Abscess in the Brain, in Cases of Disease within the Ear.* By JOSEPH TOYNBEE, F.R.S. (Medical Times and Gazette, March 16th, 1861.)

When matter is pent up in one of the cavities of the ear, it gives rise to disease of the petrous portion of the temporal bone, and the formation of an abscess in the brain. Some years ago Mr. Toynbee advanced the opinion, which has since been borne out by numerous observations, that the cerebrum was the seat of the abscess when pus was confined in the tympanum; when pent up in the mastoid cells, the cerebellum was affected, and when in the vestibule, the pons varolii and the base of the brain were involved. These cases are very insidious in their progress, and it is, therefore, highly important to prevent the collection of matter, and to remove it when once formed. If, however, the case has progressed so far that neither of these measures can be adopted, our duty is to endeavor to prevent the disease advancing to the brain.

When the tympanum is affected, and its membrane has been entirely or partly destroyed, the case is most manageable, the removal of the discharge being insured by syringing with warm water. The use of the syringe must be persevered in as long as there is any discharge from the ear, inasmuch as it is liable to become inspissated, and thus offer an impediment to its own free escape. The mucous membrane of the tympanum, which furnishes the pus, is, at the same time, to be treated by local and general measures, and when its condition is ameliorated, there is no longer fear of disease in the petrous bone.

The case is rendered much more serious when matter is pent up in the tympanum or mastoid cells, without any opening in the membrane of the tympanum. In either of these cases an operation to establish an orifice in the drum is not to be thought of, as inflammation of the dura mater may be lighted up. The best means of averting mischief to the bone, and thus preventing implication of the brain or its membranes, is by the use of an issue or a seton; and in his work, "On the Diseases of the Ear," Mr. Toynbee cites a case in which the patient had symptoms of matter in the mastoid cells, and who was cured by the introduction of a seton. If pus is pent up in the mastoid cells, and the membrane of the tympanum be absent, a curved probe may be passed into the natural passage into the tympanum, and thus exit for the matter will be afforded; but the mucous membrane is usually so much thickened that the above means will fail to reach and release the pus.

